# Agreement Between Handheld and Fixed Dynamometry for Hip Strength Assessment in Young Soccer Players and Non-Soccer Controls: An Exploratory Study

**DOI:** 10.3390/jcm15218416

**Published:** 2026-10-29

**Authors:** Rafał Borkowski, Jakub Grodzki, Patrycja Adamska

**Affiliations:** Faculty of Rehabilitation, The Józef Piłsudski University of Physical Education in Warsaw, Marymoncka 34, 00-968 Warsaw, Polandpatrycja.adamska@awf.edu.pl (P.A.)

**Keywords:** Bland–Altman analysis, DynaMo, fixed dynamometry, ForceFrame, handheld dynamometry, hip muscle strength, method agreement, soccer

## Abstract

**Background/Objectives**: Handheld dynamometers are increasingly used for muscle strength assessment because of their low cost, portability, and ease of implementation in clinical and sports settings. However, it remains unclear whether measurements obtained using handheld and externally fixed dynamometers can be used interchangeably in young soccer players. This exploratory cross-sectional study compared measurements of hip adductor and abductor strength obtained using a handheld dynamometer (DynaMo) and an externally fixed dynamometer (ForceFrame) in young soccer players and non-soccer controls. **Methods**: Forty healthy men (20 U-21 soccer players and 20 non-soccer controls) were included. Isometric hip adduction and abduction strength were assessed bilaterally using DynaMo and ForceFrame during a single testing session. Agreement between measurement methods was evaluated using Bland–Altman analysis, and associations between methods were determined using Spearman’s rank correlation coefficients. **Results**: Moderate to very strong positive correlations were observed between DynaMo and ForceFrame measurements (r = 0.44–0.96), although the correlation for left hip abduction in soccer players did not reach statistical significance (*p* = 0.052). Nevertheless, Bland–Altman analysis revealed a systematic bias, with ForceFrame producing higher normalized hip strength values than DynaMo across all assessments. The mean bias ranged from 2.02 to 3.34 N/kg (95% LoA 0.21 to 4.83 N/kg) in the soccer group, and 0.92 to 2.00 N/kg (95% LoA 0.18 to 2.93 N/kg) in the non-soccer group. **Conclusions**: Although moderate to very strong correlations were observed between the two dynamometry systems, systematic differences in recorded strength values indicate that the two systems may not be considered directly interchangeable for assessing hip abductor and adductor strength. For longitudinal monitoring and clinical decision-making, repeated assessments should preferably be performed using the same measurement system, and caution is warranted when interpreting absolute hip muscle strength values. Given the exploratory design and relatively small sample, these findings should be interpreted cautiously and confirmed in larger studies.

## 1. Introduction

Epidemiological data suggest that match-related injury incidence may be higher in youth soccer players than in adult professional players [1]. Groin injuries are among the most common musculoskeletal injuries in soccer, accounting for approximately 14–19% of all reported injuries and demonstrating high recurrence rates [2,3]. Adductor-related groin pain is the most common clinical presentation, accounting for over 60% of groin injuries in soccer players [4]. These injuries frequently result in prolonged time loss from training and competition, emphasizing the importance of identifying modifiable risk factors and implementing effective prevention strategies.

The etiology of injuries is multifactorial, and injury risk cannot be adequately assessed using a single variable [5]. Emerging approaches in precision sports medicine increasingly emphasize the integration of complementary data sources to support individualized athlete assessment and management. In soccer, omics technologies are being investigated as potential sources of information on performance, injury risk, and recovery [6]. Within this broader framework, objective assessment of physical characteristics remains important for athlete screening, injury prevention, monitoring responses to therapeutic interventions, and return-to-sport assessment [7,8,9].

Hip adductor and abductor muscles play important roles in maintaining pelvic stability and contributing to lower limb alignment during dynamic activities, including running, cutting, kicking, and rapid changes in direction [10,11]. These movements are frequently performed in soccer and impose substantial mechanical demands on the hip musculature. Consequently, deficits in hip muscle strength, particularly reduced adductor strength, have been consistently identified as modifiable risk factors for groin injuries [12]. A recent meta-analysis by Quintana-Cepedal et al. [12] demonstrated that professional soccer players with lower hip adductor strength were at increased risk of developing groin pain during the competitive season. Similarly, greater hip abductor strength, together with hip adduction strength and HAGOS scores, was associated with a lower likelihood of groin injuries in professional soccer players [13].

Consequently, it is important that measurement tools used for hip muscle strength assessment provide accurate and reproducible results. Hip muscle strength can be assessed using several methods, including isokinetic dynamometers, externally fixed dynamometers (EFD), and handheld dynamometers (HHD). EFDs are measurement systems in which the force sensor is secured to an external structure, whereas HHDs are devices designed to be used either with manual stabilization or with external fixation.

Isokinetic dynamometry is widely regarded as the reference method for objective muscle strength assessment owing to its excellent measurement accuracy and reproducibility [14]. EFDs have also demonstrated excellent reliability and validity and are increasingly used as a practical alternative to isokinetic dynamometry [15,16]. However, their relatively high cost, large size, and limited portability restrict the routine use of EFDs in field-based athlete screening. In contrast, HHDs offer a practical, relatively inexpensive, and portable alternative. Although conventional HHDs are susceptible to examiner-dependent factors, such as examiner’s strength and ability to stabilize the device, external stabilization has been shown to improve measurement performance and reduce these limitations [17]. Consequently, HHDs have become widely used for hip strength assessment owing to their practicality and demonstrated reliability [17]. It remains, however, unclear whether measurements obtained with EFDs and HHDs can be used interchangeably [18]. This distinction is relevant because absolute strength values are frequently used for decision-making. Demonstrating correlation alone is insufficient if systematic differences exist between measurement systems [19].

Accordingly, this exploratory study aimed to provide preliminary evidence on the agreement between measurements of hip adductor and abductor strength obtained using an EFD and an HHD, used according to their measurement configuration, in two groups: (1) young soccer players and (2) non-soccer controls. We hypothesized that measurements obtained using the EFD and HHD would show strong positive correlation but limited agreement.

## 2. Materials and Methods

### 2.1. Study Design

An exploratory cross-sectional study was conducted in young soccer players and non-soccer controls. Hip adductor and abductor strength was assessed using an EFD and an HHD during a single testing session. The study was approved by the Senate Research Ethics Committee of the Józef Piłsudski University of Physical Education in Warsaw (approval No. SKE/0030.38.2026). All participants provided written informed consent before participation and were informed about the study objectives, potential risks and benefits, and their right to withdraw at any time without consequences.

### 2.2. Participants

Forty male participants aged 18–21 years were enrolled in the study. The soccer group consisted of 20 male U-21 soccer players who had a minimum of six years of soccer training experience, trained regularly (≥5 sessions per week), and were active members of a soccer club competing in the Central Junior League. Measurements were performed in June, toward the end of the competitive season, while the players continued their regular training.

The control group consisted of 20 healthy male students from a university of physical education who were of a similar age. They did not participate in competitive sports or regularly engage in any sports outside their university physical education classes, which were held two to three times per week, with each session lasting 90 min (3–4.5 h per week in total). Participants were recruited using convenience sampling, with recruitment announcements distributed across the university campus. Information on physical activity and previous injuries was collected during recruitment.

As this was an exploratory study, no a priori sample size calculation was performed. Consequently, the sample size was not determined a priori based on a prespecified level of precision for estimating bias and limits of agreement.

Participants in both groups were required to be free from lower limb pain on the day of testing and to have no history of lower limb injury within the preceding six months. Exclusion criteria included female sex, age under 18 or above 21 years, current lower limb injury, pelvic or lower limb surgery within the previous year, an acute groin injury within the preceding six months, and pain during strength testing. Descriptive anthropometric characteristics of the participants are presented in Table 1.

### 2.3. Measurement Protocol

**Figure 1:**
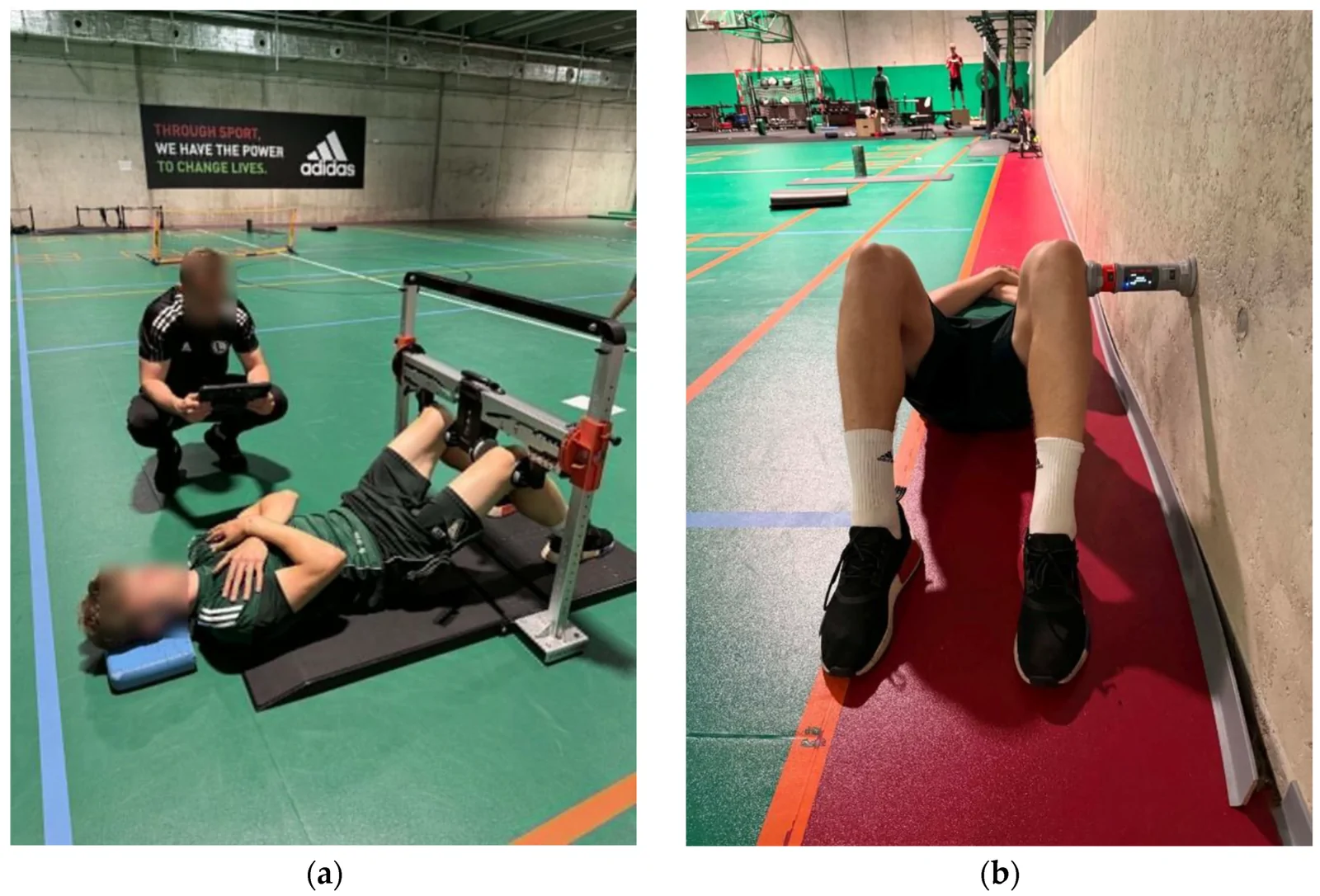
Assessment of hip abductor muscle strength using an externally fixed dynamometers- EFD (**a**) and a handheld dynamometers- HHD (**b**).

Hip adductor and abductor strength was assessed using an EFD (ForceFrame, VALD Performance, Brisbane, Australia) and an HHD (DynaMo, VALD Performance, Brisbane, Australia). Both devices were selected for comparison because they represent two approaches to strength assessment while being manufactured by the same company. For both devices, participants were tested in the supine position with the hips flexed to 45° and the knees flexed to 90°. The point of force application was standardized at the level of the knee joint. During the squeeze test, resistance was provided by the contralateral limb. During the hip abduction test, the ForceFrame system enabled simultaneous bilateral measurement of hip muscle strength (Figure 1a), whereas the HHD was positioned against a rigid wall to provide external resistance (Figure 1b).

Before testing, each participant performed a standardized warm-up consisting of running, squats, side planks, and Copenhagen adduction exercises. No separate familiarization session was conducted before testing. All participants followed the same testing order, with ForceFrame measurements performed before DynaMo measurements. With each dynamometer, participants performed three maximal voluntary isometric contractions, each lasting 3 s, with a 3-min rest interval between contractions. A 20-min rest interval was provided between testing with ForceFrame and DynaMo [20]. All measurements were performed by the same examiner (J.G.), who had four years of experience in testing soccer players. Neither the examiner nor the participants were blinded to the device being used.

A priori, it was decided that only the highest value obtained across three trials would be used for statistical analysis to represent each participant’s maximal force-generating capabilities. Hip muscle strength was recorded in newtons (N) and normalized to body mass (N/kg).

### 2.4. Statistical Analysis

All statistical analyses were performed by R.B. using JASP (version 0.98.1; JASP Team, Amsterdam, The Netherlands). Statistical significance was set at *p* < 0.05. The distribution of the continuous variables and the differences between measurements obtained using the two systems were assessed using the Shapiro–Wilk test and visually inspected using Q–Q plots. Because the primary aim of this study was to evaluate agreement and interchangeability between DynaMo and ForceFrame, Bland–Altman analysis was selected as the principal analytical approach. Bland–Altman plots were constructed to estimate the mean bias and the 95% limits of agreement (LoA) between the two measurement methods. The results of the Bland–Altman analyses were presented graphically and summarized in tables reporting the mean bias and 95% LoA. Potential proportional bias was assessed separately for each group and testing condition using linear regression, with the differences between ForceFrame and DynaMo measurements as the dependent variable and the corresponding means of the two measurements as the predictor variable. The regression slope, its 95% confidence interval, and the associated *p*-value were used to assess the presence of proportional bias. To account for multiple comparisons across the eight regression analyses, the Bonferroni correction was applied, resulting in an adjusted significance threshold of *p* < 0.00625. The strength of association between the two measurement methods was additionally quantified using Spearman’s rank correlation coefficient. The magnitude of the correlation coefficient was interpreted as follows: 0.00–0.10, negligible; 0.10–0.39, weak; 0.40–0.69, moderate; 0.70–0.89, strong; 0.90–1.00, very strong [21]. To compare agreement between groups, differences between ForceFrame and DynaMo measurements were calculated for each participant and testing condition (adduction and abduction). Differences were summarized using medians and interquartile ranges (IQRs) and compared between the soccer and non-soccer groups using the Mann–Whitney U test. The Bonferroni correction was applied to account for multiple comparisons across the four testing conditions, resulting in an adjusted significance threshold of *p* < 0.0125. Effect sizes were expressed as rank-biserial correlations.

## 3. Results

### 3.1. Correlation Between the Measurement Methods

Positive correlations were observed between measurements obtained using ForceFrame and DynaMo for hip adduction and abduction in both groups (Table 2). In the soccer group, moderate and statistically significant correlations were observed for right and left hip adduction and right hip abduction, whereas the correlation for left hip abduction did not reach statistical significance (*p* = 0.052). In the non-soccer group, moderate to very strong and statistically significant positive correlations were found for all assessments.

### 3.2. Agreement Between the Measurement Methods

**Figure 2:**
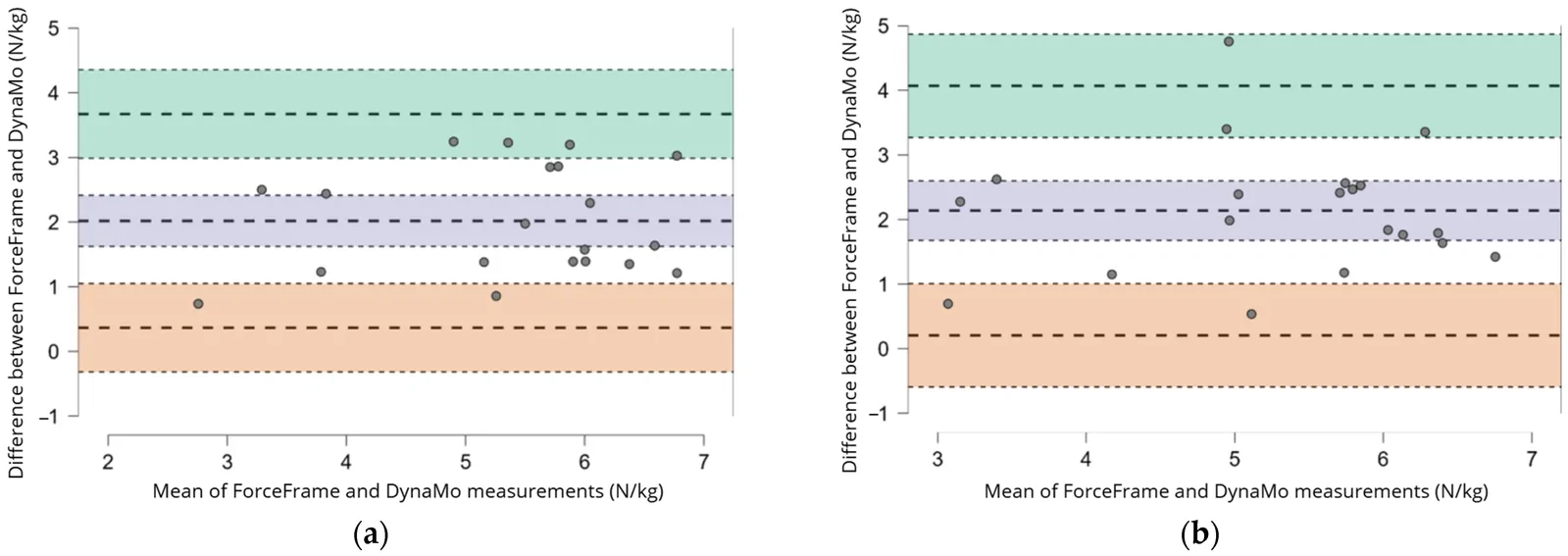

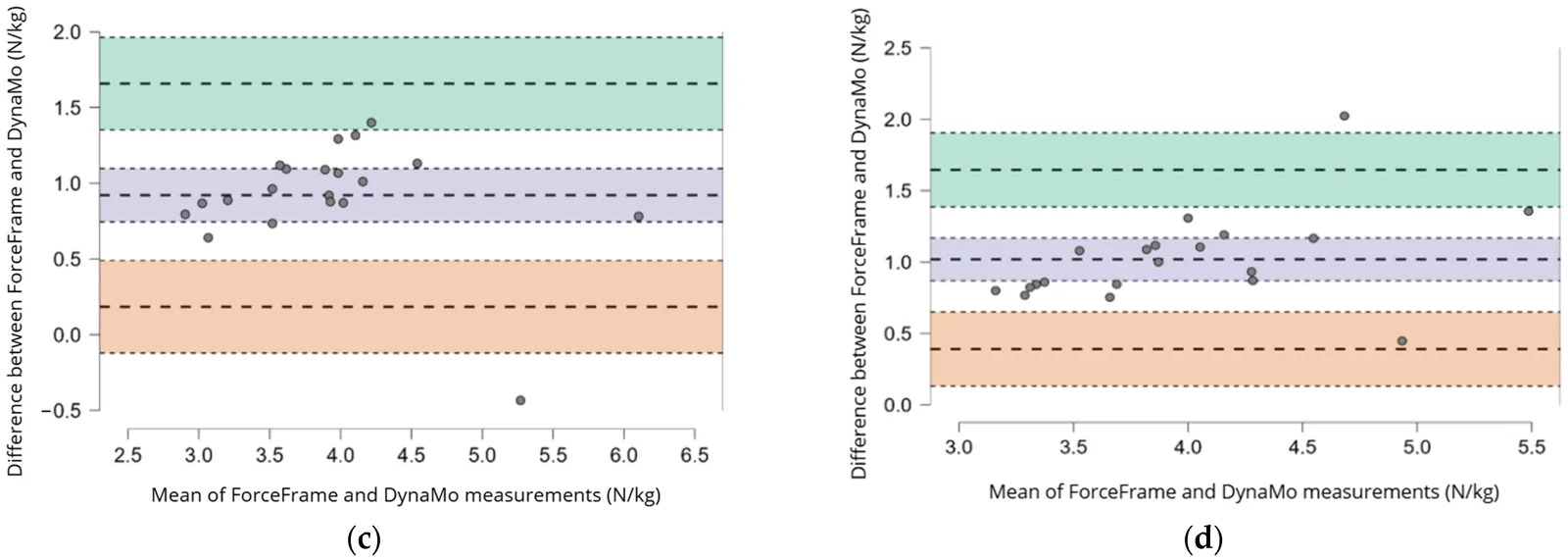
Bland–Altman plots illustrating the agreement between ForceFrame and DynaMo measurements of hip adduction strength. (**a**) Soccer group, right hip. (**b**) Soccer group, left hip. (**c**) Non-soccer group, right hip. (**d**) Non-soccer group, left hip. The central dashed line indicates the mean difference (bias), whereas the upper and lower dashed lines represent the 95% limits of agreement (LoA). The shaded areas indicate the corresponding 95% confidence intervals for the bias and the upper and lower LoA. Differences were calculated as ForceFrame—DynaMo. Each dot represents an individual participant’s mean of the ForceFrame and DynaMo measurements (x-axis) plotted against the difference between the two measurements (y-axis).

**Figure 3:**
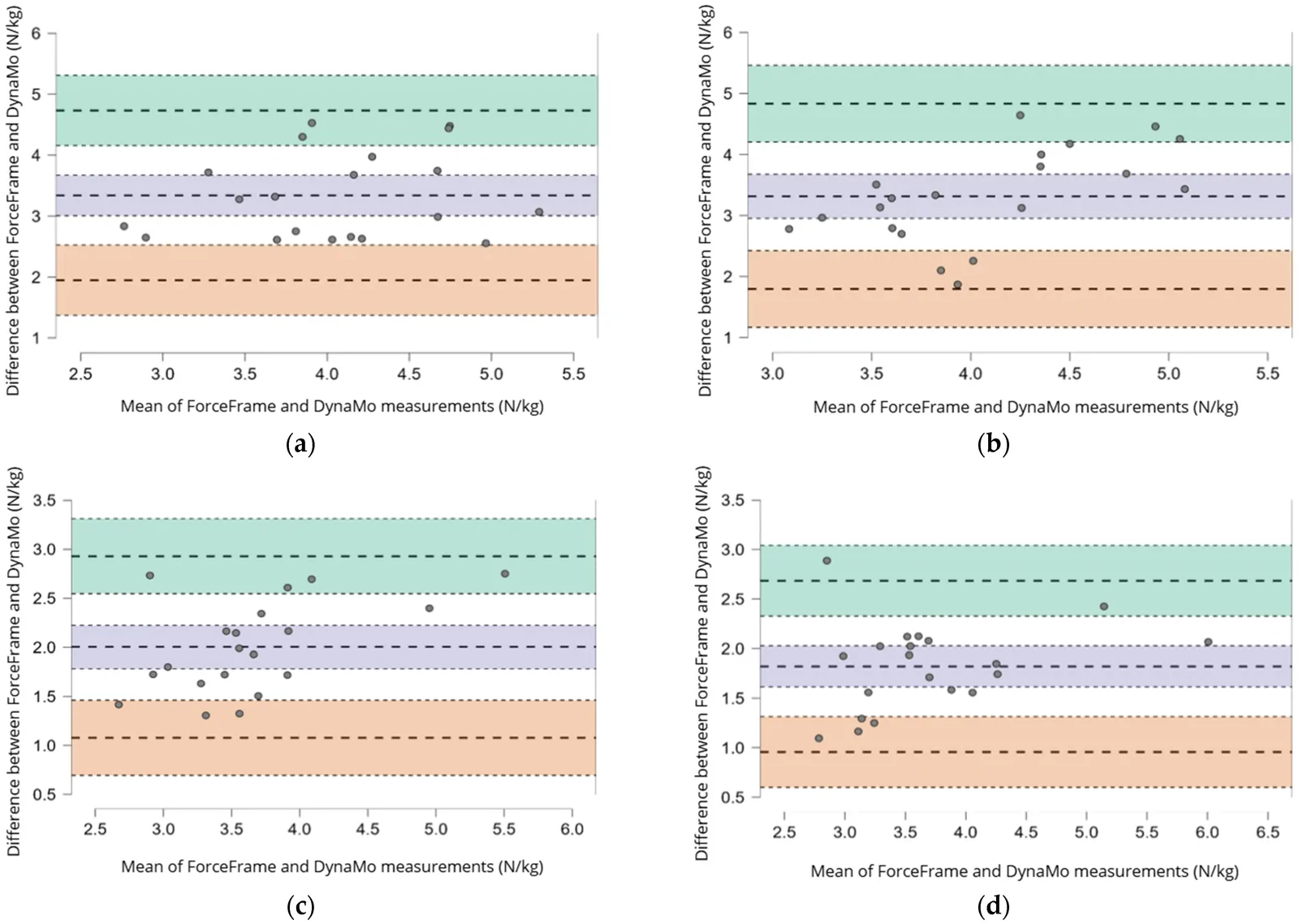
Bland–Altman plots illustrating the agreement between ForceFrame and DynaMo measurements of hip abduction strength. (**a**) Soccer group, right hip. (**b**) Soccer group, left hip. (**c**) Non-soccer group, right hip. (**d**) Non-soccer group, left hip. The central dashed line indicates the mean difference (bias), whereas the upper and lower dashed lines represent the 95% limits of agreement (LoA). The shaded areas indicate the corresponding 95% confidence intervals for the bias and the upper and lower LoA. Differences were calculated as ForceFrame—DynaMo. Each dot represents an individual participant’s mean of the ForceFrame and DynaMo measurements (x-axis) plotted against the difference between the two measurements (y-axis).

Bland–Altman analysis demonstrated a positive bias for ForceFrame relative to DynaMo across all hip adduction and abduction strength assessments (Table 3; Figure 2 and Figure 3). In the soccer group, mean differences ranged from 2.02 to 3.34 N/kg, while the 95% LoA ranged from 0.21 to 4.83 N/kg. In the non-soccer group, mean differences ranged from 0.92 to 2.00 N/kg, while the 95% LoA ranged from 0.18 to 2.93 N/kg.

The Shapiro–Wilk test and Q–Q plots indicated deviations from normality in three of the eight group-by-condition combinations. Nominally statistically significant proportional bias was observed for left hip abduction in the soccer group (β = 0.76, 95% CI: 0.23–1.29, *p* = 0.007) and right hip abduction in the non-soccer group (β = 0.38, 95% CI: 0.08–0.68, *p* = 0.017). No evidence of proportional bias was found for the remaining testing conditions. However, neither result remained statistically significant after Bonferroni correction for eight comparisons.

### 3.3. Between-Group Comparison of Measurement Systems

The between-group comparison showed significantly greater differences between ForceFrame and DynaMo measurements in the soccer group than in the non-soccer group across all four testing conditions (all *p* < 0.001). The magnitude of these differences and the corresponding effect sizes are presented in Table 4.

## 4. Discussion

The present exploratory study compared measurements obtained using an HHD (DynaMo) and an EFD (ForceFrame) in young soccer players and non-soccer controls. Moderate to very strong correlations were observed between methods. Bland–Altman analysis revealed a systematic positive bias, with ForceFrame consistently producing higher values than DynaMo. These findings indicate that while both devices measure similar constructs, they should not be considered directly interchangeable. Therefore, the initial hypothesis was only partially confirmed.

### 4.1. Correlation Versus Agreement

Bland–Altman analysis revealed a consistent tendency for ForceFrame to record higher hip muscle strength values than DynaMo across all assessments, despite moderate to very strong positive correlations observed between devices. Although measurements obtained using the two systems were positively associated, the systems cannot be assumed to measure hip muscle strength equivalently, particularly given the systematic differences in the absolute values they produce [22]. Furthermore, the correlation for left hip abduction in the soccer group did not reach statistical significance.

To the best of our knowledge, no population- and task-specific minimal clinically important difference (MCID) or other predefined criterion for clinically acceptable between-device differences was identified for hip adductor and abductor strength in young male soccer players or similarly aged healthy individuals. A recent study reported an anchor-based MCID of approximately 8.2% for hip abductor strength measured with a handheld dynamometer. However, this estimate was derived from older adults following pertrochanteric fracture and used a different testing protocol, and therefore cannot be directly applied to the present population or measurement conditions [23]. Consequently, the clinical significance of the observed bias remains uncertain.

### 4.2. ForceFrame Recorded Higher Values than DynaMo

ForceFrame consistently produced higher strength values than DynaMo. This may be related to differences in the mechanical and stabilization characteristics of the two measurement configurations. Previous work has shown that differences in stabilization methods can produce systematic differences in measured strength despite excellent reliability [24]. Consequently, at higher force outputs, relatively small differences in stabilization [25], force transmission [26], or device positioning [24] may become magnified. Externally fixed design of ForceFrame provides a highly standardized testing configuration that may reduce variability in device positioning and help maintain a consistent point of force application across participants. In contrast, despite careful adherence to the testing protocol, slight differences in HHD placement relative to anatomical landmarks and participants’ positioning against the wall may be unavoidable because of individual differences in limb morphology and the examiner’s manual positioning. Such variations may alter the effective lever arm and, consequently, influence the measured force. Second, unlike HHDs, EFDs minimize movement of the measurement apparatus during maximal isometric contractions. This could potentially reduce energy dissipation and allow a more complete transfer of force to the load cell. In contrast, HHDs may be more susceptible to small changes in device positioning or subtle movements at the interface between the limb and the dynamometer, even when external stabilization is used [24]. Although these factors were not directly measured in the present study, they represent a plausible explanation for the observed systematic bias. D’Mello et al. [25] reached similar conclusions when comparing results obtained using EFD and HHD. The authors attributed the higher values obtained using the ForceFrame to improved stabilization during testing.

Previous studies have also investigated the reliability of each measurement system separately. Collings et al. [27] reported good-to-excellent between-day reliability for DynaMo measurements of hip adduction and abduction strength, with ICC values of 0.96 and 0.88, respectively. Similarly, Rago et al. [28] evaluated different protocols for assessing hip abduction and adduction in young male football players. Using ForceFrame, the authors reported high reliability across testing protocols, although reliability varied depending on the testing position and protocol. Nevertheless, overall reliability across protocols remained between 0.84 and 0.99. These findings provide useful context for interpreting the present results. However, reliability estimates obtained in other samples and under different testing conditions cannot be directly extrapolated to the protocol used in the present study. As reliability was not assessed in our sample, it cannot be determined to what extent measurement variability may have influenced differences observed between devices.

Another consideration is that the measurement order was not randomized, with ForceFrame assessments always preceding DynaMo assessments. Although a rest interval was provided [20], potential order effects, including familiarization, learning, fatigue, or post-activation performance enhancement, cannot be excluded. These factors may have contributed to the observed differences between devices, although their actual influence cannot be determined from the present data.

### 4.3. Between-Group Differences in Measurement Agreement

As discussed above, differences in measurement stabilization and force transmission may contribute to discrepancies between dynamometry systems [24,25,26]. This may partly explain the between-group differences in agreement between the measurement systems. Compared with the non-soccer group, soccer players generally showed weaker correlations between the measurement methods and greater differences between ForceFrame and DynaMo measurements across all four testing conditions. These differences were statistically significant and associated with large effect sizes, suggesting that the magnitude of the discrepancy between the measurement systems differed between groups in this study. Additionally, regression analyses provided no statistically significant evidence of proportional bias after correction for multiple comparisons. Although nominally significant positive associations were observed for left hip abduction in soccer players and right hip abduction in non-soccer controls, neither association remained statistically significant after the Bonferroni correction.

Moreover, previous research has demonstrated that test–retest variability in dynamometry is influenced by participant familiarization and testing experience [29,30]. A similar effect may be associated with long-term athletic training. However, this possibility needs to be investigated in future studies involving larger, more homogeneous groups. Although strength values were normalized to body mass, this procedure does not eliminate all potential effects of differences in anthropometric characteristics. Therefore, the between-group findings should be interpreted cautiously and should not be attributed solely to training status.

### 4.4. Clinical Implications

Within the broader framework of precision sports medicine, strength measurements may complement emerging biological approaches used to investigate athletic adaptation, recovery, and injury susceptibility [6]. In this context, the present findings have several practical implications for clinicians and sports practitioners. HHDs offer important advantages, including lower cost, portability, and ease of implementation, making them well suited for routine clinical practice and field-based screening. However, findings of the present exploratory study suggest that measurements obtained using the EFD and HHD may not be directly interchangeable, particularly when absolute strength values are used for longitudinal monitoring or comparison across studies. This is consistent with the systematic review by Waiteman et al. [18], which concluded that the current evidence does not support interchangeable use of EFDs and HHDs. Importantly, the observed systematic bias should not be interpreted as indicating inferior performance of the HHD. Rather, the findings provide preliminary evidence that both systems measure hip muscle strength, although the measurements obtained should be interpreted in the context of the device used.

The present study did not assess criterion validity, reliability, responsiveness, or diagnostic or prognostic utility. Therefore, no conclusions can be drawn regarding these measurement properties.

### 4.5. Strengths and Limitations

This study has several strengths that should be acknowledged. To our knowledge, it is among the first to directly compare measurements obtained using an EFD and an HHD for the assessment of hip adductor and abductor strength in young soccer players. Furthermore, agreement between devices was evaluated using Bland–Altman analysis, which provides more clinically relevant information regarding interchangeability than correlation analysis alone. Standardized testing procedures, identical participant positioning, and the inclusion of both soccer and non-soccer individuals further strengthen the methodological rigor of the study.

Nevertheless, several limitations should be considered when interpreting the findings.

First, the exploratory design and relatively small sample size limit the generalizability of the findings and the precision of the estimated bias and LoA. In addition, no a priori sample size calculation was performed to determine the number of participants required to estimate agreement with a prespecified level of precision. Therefore, the Bland–Altman results should be interpreted cautiously, and larger studies are warranted to obtain more precise estimates of agreement.

Second, the study included only young male participants, which limits the generalizability of the findings to female athletes, older individuals, and clinical populations. In addition, the soccer players were recruited from a single sports club, meaning that potential selection bias cannot be excluded. The groups differed in age, body mass, and BMI, which may have influenced the between-group comparisons. Furthermore, detailed records of the numbers of individuals screened, excluded, or declining to participate were not collected. Consequently, potential selection bias cannot be fully assessed. In addition, although information on physical activity and previous injuries was collected, the overall physical activity level and training volume of the control group were not formally quantified. Therefore, additional participation in sports or resistance training cannot be ruled out and may have contributed to differences in physical characteristics and training exposure between groups.

Third, measurement reliability and measurement error were not assessed in the present sample. All measurements were performed during a single session, precluding assessment of test–retest reliability. Consequently, the extent to which measurement variability may have contributed to the observed between-device differences remains unknown.

Fourth, no population- and task-specific clinically acceptable threshold for between-device differences was predefined. Therefore, the clinical significance of the observed systematic bias cannot be determined.

Fifth, strength assessment was limited to isometric hip adduction and abduction performed in a single testing position. The order of testing was not randomized or counterbalanced. Potential familiarization or fatigue effects cannot be excluded. Moreover, examiner and participant blinding to the measurement device was not feasible because of the substantial differences in the size and configuration of the two dynamometry systems. Nevertheless, the lack of blinding may have introduced measurement bias and should be considered when interpreting the findings.

It remains unclear whether similar findings would be observed with other testing protocols or for different muscle groups. Consequently, larger studies involving more diverse populations and additional testing conditions are warranted to confirm and extend the present findings.

## 5. Conclusions

This exploratory study provides preliminary evidence on the agreement between an HHD (DynaMo) and an EFD (ForceFrame) for assessing hip adductor and abductor strength in young soccer players and non-soccer controls. Although moderate to very strong positive correlations were observed between measurements obtained using the two systems, Bland–Altman analysis revealed a systematic bias, with ForceFrame producing higher strength values than DynaMo. These findings indicate that the two systems cannot currently be assumed to be interchangeable for absolute strength measurements. The clinical significance of the observed differences remains uncertain because no population- and task-specific threshold for clinically acceptable between-device differences was predefined. Therefore, clinicians and researchers should consider using the same measurement system for repeated assessments and interpret device-specific values with appropriate caution. Further studies involving larger and more diverse populations are warranted to obtain more precise estimates of agreement and determine whether the observed differences are clinically meaningful.

## Figures and Tables

**Table 1:** Participants’ characteristics. Values are presented as mean ± standard deviation (SD). Between-group comparisons were performed using the Mann–Whitney U test. *p*-values are reported for each comparison.

Group	Soccer Group	Non-Soccer Group	*p*
Age [years]	18.75 ± 0.72	19.55 ± 0.51	<0.001
Body mass [kg]	75.95 ± 5.62	80.25 ± 6.41	0.020
Height [cm]	182.30 ± 6.28	181.10 ± 5.45	0.674
BMI [kg/m^2^]	22.85 ± 1.18	24.45 ± 1.24	<0.001

**Table 2:** Spearman’s rank correlation coefficients between measurements obtained using the ForceFrame and DynaMo systems for hip adduction and abduction in the soccer and non-soccer groups. Correlation coefficients (rho), corresponding *p*-values, and 95% confidence intervals are presented.

Group	Movement	Side	Spearman’s Rho	*p*	95% CI
Soccer	Add	Right	0.62	0.005	[0.24, 0.83]
		Left	0.59	0.007	[0.21, 0.82]
	Abd	Right	0.55	0.012	[0.14, 0.80]
		Left	0.44	0.052	[−0.01, 0.74]
Non-soccer	Add	Right	0.90	<0.001	[0.77, 0.96]
		Left	0.96	<0.001	[0.89, 0.98]
	Abd	Right	0.66	0.002	[0.31, 0.85]
		Left	0.81	<0.001	[0.58, 0.92]

CI—confidence intervals; Add—adduction; Abd—abduction.

**Table 3:** Bland–Altman analysis of agreement between ForceFrame and DynaMo measurements of hip adduction and abduction strength in the soccer and non-soccer groups. Values are presented as the mean difference (bias) and the 95% limits of agreement. Values in square brackets indicate 95% confidence intervals.

Group	Movement	Side	Bias [95% CI] (N/kg)	95% LoA [95% CI] (N/kg)
Soccer	Add	Right	2.02 [1.62, 2.41]	0.37 [−0.32, 1.05] to 3.67 [2.99, 4.36]
		Left	2.14 [1.68, 2.60]	0.21 [−0.59, 1.01] to 4.07 [3.27, 4.87]
	Abd	Right	3.34 [3.01, 3.67]	1.95 [1.37, 2.52] to 4.73 [4.16, 5.31]
		Left	3.31 [2.95, 3.68]	1.80 [1.17, 2.42] to 4.83 [4.21, 5.46]
Non-soccer	Add	Right	0.92 [0.74, 1.10]	0.18 [−0.12, 0.49] to 1.66 [1.35, 1.96]
		Left	1.02 [0.87, 1.17]	0.39 [0.13, 0.65] to 1.65 [1.39, 1.91]
	Abd	Right	2.00 [1.78, 2.22]	1.08 [0.69, 1.46] to 2.93 [2.55, 3.31]
		Left	1.82 [1.61, 2.03]	0.96 [0.60, 1.31] to 2.68 [2.33, 3.04]

LoA—limits of agreement; CI—confidence interval.

**Table 4:** Between-group comparison of differences between ForceFrame and DynaMo measurements of hip adduction and abduction strength. Values are presented as median and interquartile range (IQR), *p*-values, and rank-biserial correlations (r). * indicates statistical significance after Bonferroni correction.

Movement	Side	Group	Median [IQR] (N/kg)	*p*	r
Add	Right	Soccer	1.81 [1.48]	<0.001 *	0.79
		Non-soccer	0.94 [0.25]		
	Left	Soccer	2.13 [0.95]	<0.001 *	0.74
		Non-soccer	0.97 [0.29]		
Abd	Right	Soccer	3.17 [1.15]	<0.001 *	0.90
		Non-soccer	1.96 [0.66]		
	Left	Soccer	3.31 [1.06]	<0.001 *	0.91
		Non-soccer	1.89 [0.51]		

Add—adduction; Abd—abduction. *—significant difference between groups.

## Data Availability

The raw data supporting the findings of this study are available from the corresponding author upon reasonable request.

## Abbreviations

The following abbreviations are used in this manuscript:

BMI	Body Mass Index
EFD	Externally fixed dynamometer
HHD	Handheld dynamometer
LoA	Limits of Agreement
